# Effect of Acute Nutritional Ketosis on Circulating Levels of Growth Differentiation Factor 15: Findings from a Cross-Over Randomised Controlled Trial

**DOI:** 10.3390/biom14060665

**Published:** 2024-06-06

**Authors:** Sanjali Charles, Yutong Liu, Sakina H. Bharmal, Wandia Kimita, Maxim S. Petrov

**Affiliations:** School of Medicine, University of Auckland, Auckland 1010, New Zealand

**Keywords:** growth differentiation factor 15, appetite, eating behaviour, ketosis, β-hydroxybutyrate, nutrition, prediabetes

## Abstract

Exogenous supplementation with ketone beverages has been shown to reduce plasma glucose levels during acute nutritional ketosis. It remains to be investigated whether growth differentiation factor 15 (GDF-15)—an anorexigenic hormone—is involved in this process. The aim was to investigate the effect of a ketone ester beverage delivering β-hydroxybutyrate (KEβHB) on plasma levels of GDF-15, as well as assess the influence of eating behaviour on it. The study was a randomised controlled trial (registered at clinicaltrials.gov as NCT03889210). Individuals were given a KEβHB beverage or placebo in a cross-over fashion. Blood samples were collected at baseline, 30, 60, 90, 120, and 150 min after ingestion. Eating behaviour was assessed using the three-factor eating questionnaire. GDF-15 levels were not significantly different (*p* = 0.503) after the KEβHB beverage compared with the placebo. This finding remained consistent across the cognitive restraint, emotional eating, and uncontrolled eating domains. Changes in the anorexigenic hormone GDF-15, irrespective of eating behaviour, do not appear to play a major role in the glucose-lowering effect of exogenous ketones.

## 1. Introduction

Based on current trends, it is estimated that approximately 20% of the world’s population will have obesity by 2025 [1,2]. The negative implications of high obesity rates cannot be overstated, especially because it is also a risk factor for non-communicable diseases such as diabetes mellitus [1]. Obesity is characterised by an excessive accumulation of body fat due to an energy imbalance where consumption outweighs expenditure [3]. Extensive research has been conducted to elucidate pathways in the brain that control satiety and hunger [3]. This knowledge has opened up new avenues for understanding individuals’ eating behaviour and helping reduce the burden of obesity [3]. The area postrema—a circumventricular organ located in the hindbrain at the base of the fourth ventricle—plays a key role in integrating and transmitting chemical and neural signals to the brain regions that control feeding [4]. Receptors for various molecules are expressed on neurons located in the area postrema, including the glial cell-derived neurotrophic family receptor α-like (GFRAL) that binds specifically to growth differentiation factor 15 (GDF-15) [5]. GDF-15 is a member of the transforming growth factor-β superfamily [5]. Experimental murine studies have shown that food intake is reduced via GDF-15-GFRAL-RET signalling in the area postrema [5,6,7]. While GDF-15 levels have been significantly associated with obesity, it has been suggested that this increase in GDF-15 is likely not causing the disease but compensating for it by attempting to reduce food intake [6,8]. Native GDF-15 has a short half-life, which precludes it from being a viable therapeutic option [9]. Therefore, recombinant GDF-15 molecules have been developed with better pharmacokinetic properties and efficacy [9]. Recombinant GDF-15 molecules administered subcutaneously to obese mice and monkeys were shown to reduce food intake and glucose levels and improve insulin sensitivity [9,10]. Further, elevated GDF-15 levels stimulated an increase in hepatic triglyceride metabolism, leading to a decrease in plasma triglycerides [11]. These findings from preclinical studies suggest that targeting GDF-15 signalling pathways may have potential in the fields of obesity and metabolic health [6,8].

GDF-15 analogues for use in human studies are still under development [6,8]. Therefore, alternative methods that increase circulating levels of GDF-15 for better metabolic health outcomes may need to be explored. One possible way to increase the levels of GDF-15 is through a high-fat, low-carbohydrate ketogenic diet [12]. Ketogenic diets (compared with the normal chow diet) were shown to significantly increase serum GDF-15 levels in mice [12]. This diet induces the state of ketosis in the body, making ketone bodies (β-hydroxybutyrate, acetone, and acetoacetate) the primary source of energy [13]. The eating behaviour of people on a ketogenic diet can be assessed using the three-factor eating questionnaire (TFEQ), which measures cognitive restraint, emotional eating, and uncontrolled eating [14]. It was shown that uncontrolled eating and emotional eating decreased following a ketogenic diet administered for 12 months, suggesting that nutritional ketosis may change eating behaviour [14]. There is an unmet need to clarify whether GDF-15 plays a role in causing this change, given its role in reducing food intake. It can take weeks to reach moderate nutritional ketosis (0.5–5 nmol/L) on a ketogenic diet, and adherence to this diet can be difficult, resulting in the development of alternative methods to reach ketosis [15]. This includes acute nutritional ketosis, where exogenous ketones are ingested and the peak state of ketosis is reached within 30–60 min [15]. The Cross-over randomisEd Trial of β-hyroxybUtyrate in prediabeteS (CETUS) showed that the ingestion of a ketone ester beverage delivering β-hydroxybutyrate (KEβHB) reduced the levels of glucose and triglycerides in individuals with prediabetes [15,16]. However, there is a gap in the literature in regard to the effect exogenous ketones have on GDF-15 levels. Given that it is an anorexigenic hormone, investigating this effect may prove beneficial in further assessing the utility of acute nutritional ketosis in treating obesity and hyperglycaemia.

The primary aim of the present study was to investigate the effect of the KEβHB beverage (versus placebo) on GDF-15 levels. The secondary aim was to investigate whether eating behaviour impacted the levels of GDF-15 during nutritional ketosis. The tertiary aim was to assess the association between GDF-15 levels and changes in glucose and triglyceride levels during nutritional ketosis.

## 2. Materials and Methods

### 2.1. Study Design and Population

The study was run at the University of Auckland (New Zealand) and was part of the CETUS project. The project was a randomised controlled trial that primarily investigated the changes in the glucoregulatory response to a KEβHB beverage in individuals with prediabetes [15]. The Health and Disability Ethics Committee assessed and approved the project (18/NTB/161). It was conducted in accordance with the standards set by the Declaration of Helsinki and prospectively registered at clinicaltrials.gov (NCT03889210). The present study reports on a secondary outcome of the project, specifically plasma levels of GDF-15.

Adult individuals (≥18 years) were included in the study if they met the criteria for prediabetes diagnosis outlined by the American Diabetes Association (glycated haemoglobin (HbA1c) between 5.7% and 6.4% (39–47 mmol/mol) and/or fasting plasma glucose (FPG) between 100 and 125 mg/dL) [17]. Individuals were excluded if they took ketone supplements or followed a ketogenic diet, were diagnosed with diabetes, had received glucose-lowering medication, performed intensive physical training or participated in competitive sports, had malignancy, underwent bariatric or gastrointestinal surgery, had a cognitive disability, or had poor venous access for blood sampling at the time of the study. Participants were randomised into two sequences in a cross-over fashion. The research nurse and study participants were blinded to the sequence of allocation.

### 2.2. Study Protocol

For a total of 24 h prior to the clinic visit, participants were asked to start a food log (that aided them in consuming similar meals before both visits), undertake an overnight fast of 8–10 h, and refrain from exercise and alcohol consumption. Six blood samples were taken during each clinical visit. The first sample was collected prior to receiving the intervention, which was a 100 mL drink comprising water, a commercially available FDA-approved monoester of the ketone β-hydroxybutyrate (R-3-hydroxybutryl-1,3-hydroxybutyrate; ∆G^®^, T∆S Ltd., Oxford, UK; 4.4 mmol/kg of D-β-hydroxybutyrate—equivalent to 1.05 mL/kg and 1.9 kcal/kg of individual body weight). The placebo was a 100 mL beverage comprising water, malic acid (BioTrace^®^, Auckland, New Zealand), and arrowroot (M^c^Kenzie’s^®^, Altona, Australia). Blood samples were taken 30, 60, 90, 120, and 150 min after ingestion. Participants returned for a second clinic visit after a washout period of 7–10 days and were provided with the alternate beverage.

### 2.3. Laboratory Measurements

β-hydroxybutyrate levels were measured on whole blood immediately after collection using a handheld ketone meter and FreeStyle Optimum β-ketone test strip (Abbott Laboratories, Chicago, IL, USA). The blood samples were collected in EDTA and lithium heparin tubes (BD Vacutainer^®^; Becton, Dickinson and Company, Franklin Lakes, NJ, USA). They were then centrifuged at 4000× *g* for 5 min and separated into plasma aliquots to be stored at −80 °C. GDF-15 plasma levels were measured using a human enzyme-linked immunosorbent assay (ELISA) kit (intra-assay coefficient of variation = 4.3%, and inter-assay coefficient of variation = −1.6%) (Ansh Laboratories, Webster, TX, USA). Blood samples were also sent to LabPlus, a tertiary medical laboratory at Auckland City Hospital (New Zealand), to measure plasma glucose and triglyceride levels [15,16].

### 2.4. Anthropometric Measurements

To measure body mass index (BMI) (kg/m^2^), a digital stadiometer (Health-o-meter^®^/Pelstar LLC^©^, McCook, IL, USA) was used. Hip circumference (cm^3^) and waist circumference (cm^3^) were measured in the horizontal plane over the participant’s clothing [18].

### 2.5. Assessment of Eating Behaviour

During a clinic visit, participants completed the TFEQ to assess their eating behaviour. The version used in this study was the TFEQ-R18, which consists of 18 different questions covering three domains (cognitive restraint, emotional eating, and uncontrolled eating) and was validated for use in the general population [19]. Cognitive restraint refers to the tendency to limit food intake regardless of hunger or satiety. Emotional eating refers to the tendency to increase food intake to deal with negative emotions. Uncontrolled eating refers to the tendency to increase food intake while feeling unable to regulate it [20]. The answers participants provided to each question were assigned a score from 1 to 4. There were six, three, and nine questions in the cognitive restraint, emotional eating, and uncontrolled eating domains, respectively [19].

### 2.6. Power Calculation

The sample size of the present study was determined based on the primary endpoint—change in plasma glucose [15]. Specifically, it was hypothesised that study participants would have a 15% decrease in glucose levels following ingestion of the KEβHB drink. It was determined that a sample size of 15 individuals is required to detect (with 80% statistical power) a difference in effect size of 0.88 at an α-level of 0.05. A total of 18 individuals were recruited to account for a 20% dropout or missing samples.

### 2.7. Statistical Analysis

Prism software version 9 (GraphPad Company, Boston, MA, USA) was used to conduct all statistical analyses. Baseline characteristics were reported as mean and standard deviation or median and interquartile range. Statistical significance was determined by a *p*-value of less than 0.05. Statistical analyses were conducted in four steps. First, to determine whether the difference in the levels of GDF-15 between the groups was statistically significant, a paired *t*-test was conducted. The levels of GDF-15 from 0 to 150 min for the KEβHB and placebo groups were log-transformed and plotted. The total area under the curve (AUC) was calculated using the trapezoidal rule. Q-Q plots were used to assess normality. Second, to determine whether there was an effect of time, treatment, or both on the levels of GDF-15, a repeated measure two-way ANOVA test was conducted. To meet the sphericity assumption, the Geisser-Greenhouse correction was used. Third, participants were stratified into low and high eating behaviour subgroups based on the median score of TFEQ for the three domains—cognitive restraint, emotional eating, and uncontrolled eating. Steps one and two of the analyses (described above) were repeated. Last, using data from the KEβHB group, a Spearman’s correlation analysis was conducted, investigating the association between the levels of GDF-15 at 30, 60, 90, 120, and 150 min and the changes in the levels of glucose and triglycerides from baseline to 30, 60, 90, 120, and 150 min.

## 3. Results

### 3.1. GDF-15 in the Study Population

**Figure 1:**
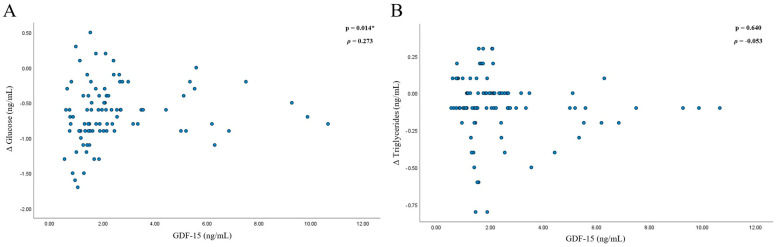
Associations between GDF-15 and changes in glucose and triglycerides. Footnotes: * indicates statistical significance (*p* < 0.05). Spearman’s correlation analysis was conducted between two variables: 1. GDF-15 levels at 30, 60, 90, 120, and 150 min; 2. change in glucose (**A**) and triglycerides (**B**) from baseline to 30, 60, 90, 120, and 150 min. Analysis was conducted on data from the KEβHB group only. Abbreviations: GDF-15 = growth differentiation factor 15; KeβHB = ketone monoester β-hydroxybutyrate.

Of the 32 individuals considered, 18 met the eligibility criteria and were randomised (12 men and 6 women). All the participants completed the study. The characteristics of the 18 study participants are presented in Table 1. The median level of GDF-15 at baseline was 2.1 ng/mL, and the interquartile range was 1.1–2.5 ng/mL. Changes in the levels of glucose from baseline to 30–150 min were significantly (*p* = 0.014, ρ = 0.273) associated with the GDF-15 levels from 30 to 150 min. Changes in the levels of triglycerides from baseline to 30–150 min were not significantly (*p* = 0.640, ρ = −0.053) associated with the levels of GDF-15 from 30–150 min (Figure 1).

### 3.2. Effect of Acute Nutritional Ketosis on Levels of GDF-15

**Figure 2:**
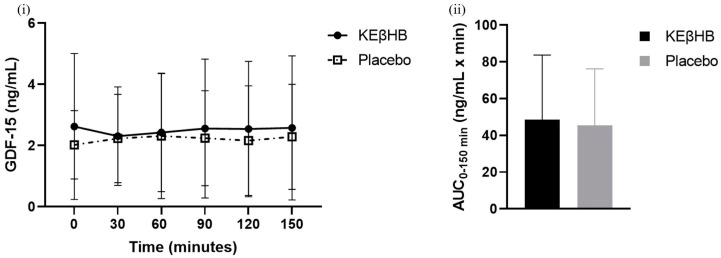
Change in the levels of GDF-15 after ingestion of the KEβHB and placebo beverages. Footnotes: mean absolute concentrations of GDF-15 from 0 to 150 min are presented in panel (**i**). Log-transformed AUCs of GDF-15 are presented in panel (**ii**). In both panels, error bars represent standard deviation. Abbreviations: AUC = area under the curve; GDF-15 = growth differentiation factor 15; KEβHB = ketone monoester β-hydroxybutyrate.

The difference between the total AUCs for the KEβHB (48.54 ± 35.21 ng/mL × min) and placebo (45.50 ± 30.72 ng/mL × min) drinks was not statistically significant (*p* = 0.503; d = 0.09) (Table 2). The effect of time alone (*p* = 0.962), treatment alone (*p* = 0.687), or interaction between time and treatment (*p* = 0.812) on the levels of GDF-15 was not statistically significant (Figure 2).

### 3.3. Role of Eating Behaviour

**Figure 3:**
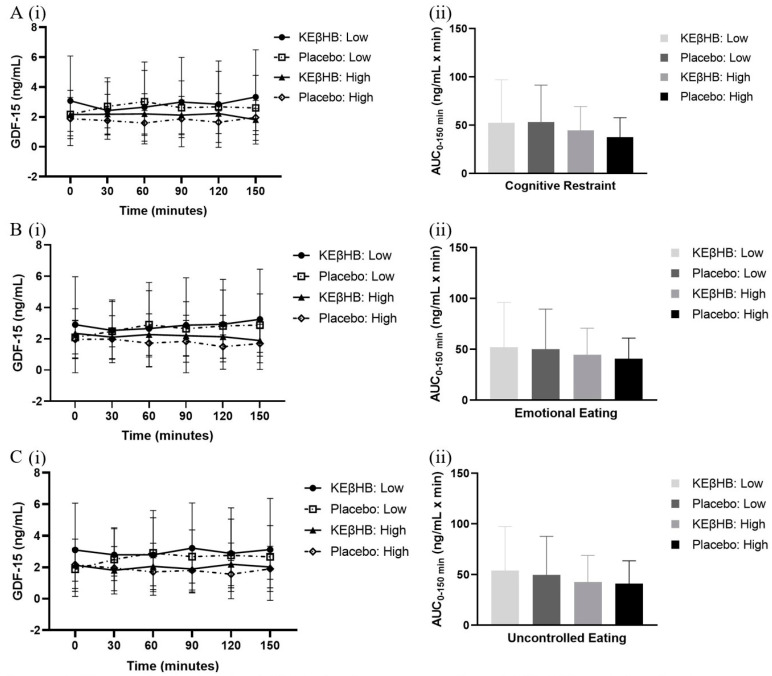
Change in the levels of GDF-15 after ingestion of KEβHB and placebo beverages according to the eating behaviour domains. Footnotes: mean absolute concentrations of GDF-15 from 0 to 150 min and log-transformed AUCs of GDF-15 grouped by high and low uncontrolled eating categories are presented in panels (**A**(**i**,**ii**)), respectively. Mean absolute concentrations of GDF-15 from 0 to 150 min and log-transformed AUCs of GDF-15 grouped by high and low cognitive restraint categories are presented in panels (**B**(**i**,**ii**)), respectively. Mean absolute concentrations of GDF-15 from 0 to 150 min and log-transformed AUCs of GDF-15 grouped by high and low EE categories are presented in panels (**C**(**i**,**ii**)), respectively. In all panels, error bars represent standard deviation. Abbreviations: AUC = area under the curve; GDF-15 = growth differentiation factor 15; KEβHB = ketone monoester β-hydroxybutyrate.

In participants with low cognitive restraint scores, the difference between the total AUCs for the KEβHB (52.51 ± 44.50 ng/mL × min) and placebo (53.48 ± 38.10 ng/mL × min) drinks was not statistically significant (*p* = 0.850; d = 0.02) (Table 3). The effect of time alone (*p* = 0.832), treatment alone (*p* = 0.996), or the interaction between time and treatment (*p* = 0.093) on the levels of GDF-15 was not statistically significant (Figure 3). In participants with high cognitive restraint scores, the difference between the total AUCs for the KEβHB (44.56 ± 24.87 ng/mL × min) and placebo (37.52 ± 20.25 ng/mL × min) drinks was not statistically significant (*p* = 0.372; d = 0.31) (Table 3). The effect of time alone (*p* = 0.923), treatment alone (*p* = 0.454), or the interaction between time and treatment (*p* = 0.873) on the levels of GDF-15 was not statistically significant (Figure 3).

In participants with low emotional eating scores, the difference between the total AUCs for the KEβHB (52.31 ± 43.85 ng/mL × min) and placebo (50.22 ± 39.29 ng/mL × min) drinks was not statistically significant (*p* = 0.640; d = 0.05) (Table 3). The effect of time alone (*p* = 0.338), treatment alone (*p* = 0.891), or the interaction between time and treatment (*p* = 0.937) on the levels of GDF-15 was not statistically significant (Figure 3). In participants with high emotional eating scores, the difference between the total AUCs for the KEβHB (44.76 ± 26.07 ng/mL × min) and placebo (40.78 ± 20.27 ng/mL × min) drinks was not statistically significant (*p* = 0.634; d = 0.17) (Table 3). The effect of time alone (*p* = 0.644), treatment alone (*p* = 0.402), or the interaction between time and treatment (*p* = 0.538) on the levels of GDF-15 was not statistically significant (Figure 3).

In participants with low uncontrolled eating scores, the difference between the total AUCs for the KEβHB (54.20 ± 43.32 ng/mL × min) and placebo (49.81 ± 38.19 ng/mL × min) drinks was not statistically significant (*p* = 0.260; d = 0.11) (Table 3). The effect of time alone (*p* = 0.191), treatment alone (*p* = 0.740), or the interaction between time and treatment (*p* = 0.277) on the levels of GDF-15 was not statistically significant (Figure 3). In participants with high uncontrolled eating scores, the difference between the total AUCs for the KEβHB (42.87 ± 26.17 ng/mL × min) and placebo (41.18 ± 22.47 ng/mL × min) drinks was not statistically significant (*p* = 0.845; d = 0.07) (Table 3). The effect of time alone (*p* = 0.524), treatment alone (*p* = 0.817), or the interaction between time and treatment (*p* = 0.102) on the levels of GDF-15 was not statistically significant (Figure 3).

## 4. Discussion

The present study was the first randomised controlled trial that examined the effect of exogenous ketone supplementation on GDF-15 levels in participants with prediabetes. We found that the effect of the KEβHB beverage on the levels of GDF-15 was not statistically significant compared with the placebo beverage. This finding held true when participants were stratified according to their eating behaviour into cognitive restraint, emotional eating, and uncontrolled eating domains. Correlation analyses showed that the levels of GDF-15 were significantly associated with the changes in the levels of glucose from baseline and not significantly associated with the changes in the levels of triglycerides from baseline during ketosis.

Ketogenic diets have gained traction as an intervention for obesity. There is evidence that this diet not only reduces body weight but also improves glycaemic control. This is a result of the body entering a state of ketosis, where fat-derived molecules (i.e., ketone bodies) become the primary source of energy. Ketosis can also be effectively achieved by the ingestion of ketone supplements [15]. These easily consumable supplements have consistently been shown to readily reduce plasma glucose levels, which suggests that they may be a more accessible treatment for people with obesity and diabetes compared with a ketogenic diet. There are two important advantages that ketosis offers in comparison with other energy-generating mechanisms. First, it prevents the degradation of skeletal muscle. The body does not have protein stores, unlike the adipose tissue that stores fat. Most protein is stored in muscles. Therefore, when the non-carbohydrate substrates are amino acids (during gluconeogenesis), they are derived from the breakdown of skeletal muscle via proteolysis to generate fuel [21]. There is evidence that ketones may promote the synthesis of proteins in muscles [22,23]. The evolutionary adaptation of preferentially using fat stores over protein stores as a fuel source prevents the breakdown of protein stores [24]. Second, ketosis provides a fuel source that is used by the brain. Under most physiological conditions, the main source of energy for the brain is glucose. However, during starvation, while vital organs such as the liver are able to use free fatty acids as a fuel source, the brain preferentially metabolises ketones rather than free fatty acids for energy. Free fatty acids have to undergo β-oxidation to generate adenosine triphosphate—the molecule that provides energy. The process of β-oxidation requires high amounts of oxygen, which produces superoxide chemicals that can cause neuron injury due to oxidative stress. It is believed that the brain has evolved to lower the expression of proteins involved in β-oxidation to protect it from injury. Ketones do not require β-oxidation, which is why they are preferred for use by the brain [25]. Understanding the pathways that cause the glucose-lowering effect of exogenous ketone supplementation may provide deeper insights into improved metabolic health outcomes in people in the state of ketosis. Therefore, a key characteristic of obesity—problematic eating behaviour—was considered in the present study in an effort to elucidate the physiological mechanisms behind the established glucose-lowering effect of acute nutritional ketosis and inform the future application of exogenous ketone supplements.

An absence of statistically significant changes in GDF-15 levels during acute nutritional ketosis suggests that the main effects of the KEβHB drink are not achieved through alterations in food intake. Poffe and colleagues reported that exogenous ketone supplementation reduced the levels of GDF-15 compared with the control beverage when consumed after each endurance training session over three weeks [26]. The authors showed that the physiological stress response observed in the overtraining stages was significantly less prominent in the ketone supplementation group compared with the placebo group. Protection against the stress response is likely the reason that the levels of GDF-15 were lower after ketone supplementation in that study. Also, Poffe and colleagues observed that energy intake was increased in the ketone supplementation group compared with the control group. Since the control group had higher levels of GDF-15 (an anorexigenic hormone), their food intake would be expected to be lower [26]. In the present CETUS study, participants were sedentary, and this may explain why no difference in the levels of GDF-15 was observed (i.e., the magnitude of the stress response was much lower compared with that observed during intensive training). A study in recreational female athletes, who were placed on a diet that restricted calories by 30% for four weeks and consumed a ketone supplement before every meal, also reported no statistically significant changes in GDF-15 [27]. That study also reported no changes in the circulating levels of ghrelin and leptin [27]. It is pertinent to note here that another CETUS study also found no significant effect of acute ketone supplementation on the circulating levels of leptin [28]. The utility that exogenous ketone supplements may have during weight loss interventions is likely based on their ability to reduce stress markers during exercise and prolong the period of intense activity rather than modulate appetite [26,27]. This is further confirmed by an earlier CETUS study that reported no statistically significant changes in the appetite hormones ghrelin and peptide YY and no statistically significant differences in hunger when comparing the KEBHB group and the placebo group [29]. Prior to that study, there was a discrepancy in the literature about whether hunger is reduced during acute nutritional ketosis [29,30,31]. The earlier studies that reported on significant changes in appetite after ketone supplement consumption included controls that were isocaloric dextrose solutions and, therefore, contained carbohydrates (unlike the placebo used in the CETUS project) [29]. The carbohydrates likely caused a reduction in the levels of ghrelin, resulting in the observed effect of appetite modulation [29]. The present study takes the field further by grouping the participants according to high versus low scores in three eating behaviour categories before looking at the change in levels of GDF-15. The TFEQ has been validated in the general population and has been shown to successfully identify eating behaviour [19]. Even in this subgroup analysis, no statistically significant differences in the circulating levels of GDF-15 were observed. This finding is in line with the results reported by Liu and colleagues when participants were stratified into the same three subgroups and found no statistically significant differences in levels of ghrelin, PYY, and hunger [29]. The inference made in the earlier study that the regulation of appetite is not mediating the effects observed during acute ketosis is therefore strengthened by the findings presented in this study.

GDF-15 levels in the present study were associated with changes in the levels of glucose from baseline but not with changes in the levels of triglycerides from baseline. The statistically significant reductions in glucose and triglyceride levels during ketosis were documented in earlier CETUS studies [15,16]. It is possible that the significant correlation observed in the present study exists for glucose (but not triglycerides) because acute nutritional ketosis causes greater reductions in the levels of glucose compared with triglycerides. The changes in glucose were more evenly spread and ranged from approximately +0.50 to −1.75 ng/mL, whereas the changes in triglycerides ranged from approximately +0.25 to −1.75 ng/mL, and most data points were concentrated near the 0 to −0.10 ng/mL range (Figure 2). Therefore, the trend of higher GDF-15 levels being associated with a greater reduction is more prevalent in the glucose analysis. Our findings suggest that GDF-15 might not be involved directly (but rather indirectly) in the reduction in glucose levels during acute nutritional ketosis. An earlier CETUS study showed that this effect was likely mediated by a glucose-dependent insulinotropic peptide—an incretin secreted by enteroendocrine K cells in the gut [15]. It stimulates β cells in the pancreas with a view to releasing insulin, subsequently leading to a reduction in plasma blood glucose. Further studies are warranted to investigate the role of other gut and pancreatic hormones during acute nutritional ketosis.

The present study had several limitations. First, the present study might have been underpowered to detect changes in GDF-15 as the sample size calculation was based on the primary endpoint (i.e., change in plasma glucose). However, this study may help in the design of future adequately powered randomised controlled trials that investigate GDF-15. Second, the study participants included both men and women of varied ages. GDF-15 levels increase with age, and this increase is different in men and women [30]. Also, earlier studies that investigated the levels of GDF-15 during acute nutritional ketosis had study populations made up of either men alone or women alone [31,32]. However, the cross-over design mitigated this issue because every participant was paired with their own results when comparing the levels of GDF-15 after consumption of both beverages. Third, only the acute effect of nutritional ketosis was studied in the present study, and therefore, the effect of long-term consumption of the KEβHB beverage on the circulating levels of GDF-15 remains unknown. There have been at least three earlier studies that investigated the effects of chronic ingestion of exogenous ketone supplements on plasma glucose levels [33,34,35]. All studies published to date deemed exogenous ketone supplements to be safe, with the longest study period being 4 weeks [36]. In the future, an investigation into the long-term effect of ketone drinks on GDF-15 is expected.

## 5. Conclusions

The beneficial role KEBHB-supplemented beverages have for people with prediabetes does not include an acute change in plasma levels of GDF-15. Eating behaviour does not appear to affect the levels of GDF-15 during acute nutritional ketosis. While there is no effect of acute nutritional ketosis on the circulating levels of GDF-15 in the prediabetes human model, its effect on diabetes or obesity warrants purposefully designed clinical and basic science investigations.

## Figures and Tables

**Table 1:** Characteristics of study participants.

Characteristic	Participants (*n* = 18) ^1^
Age, y	55 ± 14
Sex, *n*	
Men	12
Women	6
GDF-15, ng/mL	2.1 (1.1–2.5)
Systolic blood pressure, mmHg	135 ± 27
Diastolic blood pressure, mmHg	88 ± 13
BMI, kg/m^2^	28.4 (24.5–30.9)
Hip circumference, cm	108.5 (99.8–112.3)
Waist circumference, cm	100.0 (90.3–107.5)

Footnotes: ^1^ values are presented as means ± standard deviation or median (interquartile range). Abbreviations: BMI = body mass index; GDF-15 = growth differentiation factor 15.

**Table 2:** Differences in the levels of GDF-15 after ingestion of the KEβHB and placebo beverages.

Molecule	KEβHB (*n* = 18)	Placebo (*n* = 18)	Mean Difference (95% CI)	d (Effect Size)	*p*-Value
GDF-15 (ng/mL × min)	48.54 ± 35.21	45.50 ± 30.72	−3.04 (−12.41, 6.33)	0.09	0.503

Footnotes: levels of GDF-15 from 0 to 150 min were log-transformed, and then AUCs were calculated. A paired *t*-test was run to determine the mean difference between the AUCs of both groups, effect size, and *p*-value. The values are presented as means ± standard deviation or median (IQR). Abbreviations: AUC = area under the curve; GDF-15 = growth differentiation factor 15; KeβHB = ketone monoester β-hydroxybutyrate; IQR = interquartile range.

**Table 3:** Differences in the levels of GDF-15 after ingestion of the KEβHB and placebo beverages according to the eating behaviour domains.

Domain	Time	KEβHB (*n* = 9)	Placebo (*n* = 9)	Mean Difference (95% CI)	d (Effect Size)	*p*-Value
*Cognitive Restraint*						
Low	Baseline	0.32 ± 0.40	0.28 ± 0.25	−0.04 (−0.24, 0.15)	0.13	0.624
	Total AUC	52.51 ± 44.50	53.48 ± 38.10	0.96 (14.82, 4.94)	0.02	0.850
High	Baseline	0.26 ± 0.26	0.20 ± 0.28	−0.06 (0.36, 0.12)	0.21	0.648
	Total AUC	44.56 ± 24.87	37.52 ± 20.25	−7.05 (−24.21, 10.12)	0.31	0.372
*Emotional Eating*						
Low	Baseline	0.29 ± 0.39	0.28 ± 0.18	−0.01 (−0.23, 0.22)	0.02	0.941
	Total AUC	52.31 ± 43.85	50.22 ± 39.29	−2.09 (−12.03, 7.84)	0.05	0.640
High	Baseline	0.29 ± 0.29	0.20 ± 0.33	−0.09 (−0.34, 0.15)	0.30	0.410
	Total AUC	44.76 ± 26.07	40.78 ± 20.27	−3.99 (−22.59, 14.61)	0.17	0.634
*Uncontrolled Eating*						
Low	Baseline	0.35 ± 0.35	0.19 ± 0.29	−0.16 (−0.41, 0.10)	0.48	0.197
	Total AUC	54.20 ± 43.32	49.81 ± 38.19	−4.39 (−12.75, 3.96)	0.11	0.260
High	Baseline	0.23 ± 0.31	0.28 ± 0.24	0.06 (−0.13, 0.24)	0.20	0.512
	Total AUC	42.87 ± 26.17	41.18 ± 22.47	−1.69 (−21.02, 17.64)	0.07	0.845

Footnotes: levels of GDF-15 (ng/mL) from 0 to 150 min were log-transformed, and then AUCs were calculated. Paired *t*-tests were run to determine the mean difference between the AUCs of both pairs, effect size, and *p*-values. The values are presented as means ± standard deviation or median (IQR). Abbreviations: AUC = area under the curve; GDF-15 = growth differentiation factor 15; KEβHB = ketone monoester β-hydroxybutyrate; IQR = interquartile range.

## Data Availability

The original contributions presented in the study are included in the article, further inquiries can be directed to the corresponding author.
